# Characterization of Three Porcine *Acinetobacter towneri* Strains Co-Harboring *tet*(X3) and *bla*_OXA-58_

**DOI:** 10.3389/fcimb.2020.586507

**Published:** 2020-12-10

**Authors:** Jiangang Ma, Juan Wang, Jie Feng, Yingqiu Liu, Baowei Yang, Ruichao Li, Li Bai, Tao He, Xinglong Wang, Zengqi Yang

**Affiliations:** ^1^ College of Veterinary Medicine, Northwest A&F University, Yangling, China; ^2^ College of Food Science and Engineering, Northwest A&F University, Yangling, China; ^3^ Jiangsu Co-Innovation Center for Prevention and Control of Important Animal Infectious Diseases and Zoonoses, College of Veterinary Medicine, Yangzhou University, Yangzhou, China; ^4^ National Health Commission Key Laboratory of Food Safety Risk Assessment, Food Safety Research Unit (2019RU014) of Chinese Academy of Medical Science, China National Center for Food Safety Risk Assessment, Beijing, China; ^5^ Key Laboratory for Control Technology and Standard for Agro-product Safety and Quality, Ministry of Agriculture and Rural Affairs, Jiangsu Key Laboratory for Food Quality and Safety—State Key Laboratory Cultivation Base of Ministry of Science and Technology, Institute of Food Safety and Nutrition, Jiangsu Academy of Agricultural Sciences, Nanjing, China

**Keywords:** *Acinetobacter towneri*, *tet*(X3), IS*26*, circular intermediate, *bla*_OXA-58_

## Abstract

Tigecycline is the antibiotic of last resort for the treatment of extensively drug-resistant bacterial infections, mainly those of multidrug-resistant Gram-negative bacteria. The plasmid-mediated *tet*(X3) gene has recently been described in various pathogens that are resistant to tigecycline. We report three tigecycline-resistant *Acinetobacter towneri* strains isolated from porcine faeces in China, which all contained the *tet*(X3)-harboring plasmids. A broth microdilution method was used to examine the antimicrobial susceptibility of the isolates, and S1-Nuclease digestion pulsed-field gel electrophoresis (S1-PFGE) was used to characterize their plasmid profiles. The whole-genome sequences of the isolates were determined with the Nanopore PromethION platform. The sequence analysis indicated that the strains were *A. towneri.* They showed resistance to multiple antibiotics, and all the resistance genes were located on plasmids. The three *tet*(X3)-harboring plasmids had a similar backbone structure, and all contained *bla*_OXA-58_ with various insertion elements (IS). IS*CR2* is considered an important factor in *tet*(X3) mobilization. In addition to IS*CR2*, we demonstrate that IS*26* generates a circular intermediate containing the *tet*(X3) gene, which could increase the dissemination risk. To our knowledge, this is the first report of *tet*(X3)- and *bla*_OXA-58_-harboring plasmids in *A. towneri*. Because the IS*26* is frequently found in front of *tet*(X3), research should be directed toward the action of IS*26* in the spread of *tet*(X3).

## Introduction

Tigecycline, a broad-spectrum modified minocycline derivative, is considered a drug of last resort against multidrug-resistant (MDR) bacteria, especially carbapenem-resistant Enterobacteriaceae (CRE) (Tasina et al., 2011). However, recent studies have reported that plasmid-mediated tigecycline- resistance genes have been detected in various bacterial species. In particular, the novel tigecycline- resistance genes *tet*(X3), *tet*(X4), *tet*(X5), and *tet*(X6) have recently been discovered in Enterobacteriaceae and *Acinetobacter* from sewage, livestock and humans (He et al., 2019; Wang et al., 2019; He et al., 2020).

The *tet*(X3) gene has been identified in *Acinetobacter baumannii*, *A. schindleri*, *A. indicus*, and *Empedobacter brevis*, but limited information about the mechanisms of *tet*(X3) transmission in bacteria is available (Li et al., 2019; He et al., 2020a). It has been shown that *tet*(X3) is transferred by plasmids or transposons in a mechanism called ‘rolling-circle (RC) transposition’ *via* IS*CR2* (He et al., 2019). Moreover, plasmids carrying both *tet*(X3) and *bla*_NDM-1_ were detected in *A. indicus* in a recent study, but with no information on the plasmid containing *tet*(X3) together with other carbapenemase genes in *A. towneri* (He et al., 2020a).

In this study, we isolated plasmids carrying both *tet*(X3) and *bla*_OXA-58_ from *A. towneri* strains isolated from porcine faeces in China. We describe the whole-genome sequences of the three *A. towneri* isolates harboring *tet*(X3) and show that IS*26* plays an important role in the spread of *tet*(X3).

## Materials and Methods

### Clinical Isolates and Detection of the *tet*(X) Genes

One hundred and sixteen anal swabs from pigs were collected on a pig farm in Guangxi Province, southern China, in 2019. The samples were stored at low temperature and transported directly to the laboratory. Three *Acinetobacter* strains were isolated from three independent anal swabs and cultured on Leeds Acinetobacter Agar containing 4 mg/L tigecycline. These strains were designated GX3, GX5, and GX7. We screened for the tigecycline-resistant genes *tet*(X3), *tet*(X4), and *tet*(X5) with PCR, as described in a previous report (Ji et al., 2020).

### Antimicrobial Susceptibility Testing

The broth microdilution method was used to examine the antimicrobial susceptibility (ampicillin, amoxicillin-clavulanate, spectinomycin, tetracycline, florfenicol, sulfisoxazole, sulfamethoxazole, ceftiofur, ceftazidime, colistin, gentamicin, meropenem, and imipenem) of the isolates according to the Clinical Laboratory Standards Institute (CLSI) guidelines (CLSI, 2019), and *E. coli* ATCC 25922 was used as the quality control. The breakpoint criteria for tigecycline in *Acinetobacter* spp. was evaluated according to the EUCAST epidemiological cut-off values (https://www.mic.eucast.org/Eucast2/).

### S1-Nuclease Digestion Pulsed-Field Gel Electrophoresis (S1-PFGE)

S1-PFGE was performed to characterize the plasmid profiles in the three strains. *Salmonella* H9812 was used as the size standard. Briefly, the cultured cells (optical density at 600 nm of 1.0) were harvested and suspended in Tris–EDTA buffer (pH 8.0), and then mixed with 2% gold agarose (SeaKem^®^ Gold Agarose, Lonza, Atlanta, GA, USA) to make plugs. The plugs were treated with S1 nuclease (TaKaRa, Dalian, China) at 37°C for 15 min, and the DNA fragments were separated with the CHEF Mapper XA system (Bio-rad, USA), as previously described (Barton et al., 1995).

### Conjugation and Electrotransformation Analysis

The plasmids were extracted and used to electrotransform into the recipient strains (laboratory strains *Escherichia coli* DH5α, *E. coli* C600, and *bla*_OXA-23_-positive *A. baumannii* isolate 5AB) (He et al., 2019). The plasmid-free strains *E. coli* C600 (streptomycin resistant) and *E. coli* 26R 793 (rifampin resistant) were chosen as recipient strains for conjugation (Wang et al., 2013).

### Whole Genome Sequencing

Whole-genome sequencing (WGS) of the three *A. towneri* isolates was performed with the Nanopore PromethION platform (Biomarker Technologies, Beijing, China). The sequences were assembled with Canu v1.5. Pilon v1.22 was used to improve the draft genome assemblies by correcting bases.

### Genome Analysis

The bioinformatics analysis of these sequences was conducted at the CGE server (https://cge.cbs.dtu.dk/services/), including multi-locus sequence typing (MLST) to identify the (STs), and ResFinder to detect drug-resistance genes. The WGS annotations were designed with the Rapid Annotations using Subsystem Technology (RAST) annotation pipeline (version 2.0) (http://rast.nmpdr.org/). A phylogenetic tree based on plasmid replication initiator protein genes was constructed with the MEGA version 7.0 software using the neighbour-joining method (Kumar et al., 2016). The sequence alignment was generated with Easyfig version 2.1 (Sullivan et al., 2011).

## Results and Discussion

### Strain Identification and Antimicrobial Susceptibility Testing

A 16S rDNA PCR detection and sequencing analysis suggested that these strains belonged to the genus *Acinetobacter*. The *rpoB* gene was then used to identify the *Acinetobacter* isolates at the species level. This analysis showed that strain GX3 was identical to GX5, and when compared with *Acinetobacter* spp. from the GenBank database, it shared a high degree of nucleotide similarity (99.85%) with strain 19110F47 isolated from the faeces of swine in China (CP046045.1). The *rpoB* gene of strain GX7 shared greatest homology with that of strain 205 (97.21% identical at the nucleotide level) isolated from swine faeces in China (CP048014.1).

S1-PFGE was used to identify the plasmids in the isolates, and indicated that the GX5 and GX7 isolates both contained one large plasmid of ~180-kb in size. Strain GX3 contained two plasmids, approximately 50-kb and 150-kb in size. Electroporation and conjugation experiments showed that the tet(X3)-harboring plasmids in the three isolates could not transfer to *E. coli* DH5α, *E. coli* C600, *E. coli* 26R 793, or *A. baumannii* 5AB. This could be related to the host specificity of the plasmids.

All of these strains were resistant to ampicillin, amoxicillin-clavulanate, spectinomycin, tetracycline, florfenicol, sulfisoxazole, sulfamethoxazole, ceftiofur, ceftazidime, imipenem, and tigecycline, but were susceptible to gentamicin, meropenem and colistin. The strains also showed different minimal inhibitory concentration to enrofloxacin and ofloxacin (**Table S1**). The *tet*(X3) gene was identified in all three isolates.

### WGS Analysis

Strain GX3 contained one 2.59-Mb chromosome and two plasmids (148-kb and 54-kb). Strain GX5 contained a 2.63-Mb chromosome and a single plasmid (178-kb). Strain GX7 contained a 2.84-Mb chromosome and one plasmid (178-kb) (**Table 1**).

**Table 1:** Characterization of the *A. towneri* strains and their corresponding plasmids carrying the *tet*(X3) gene.

Strains	Species	Chromosomes(-bp)	GC contents(%)	Plasmids(-bp)	ORFs	Multiple drug resistance genes	Accession no.
GX3	*A. towneri*	2,597,534	41.48	148,166	166	*sul2*, *mph(E)*, *msr(E)*, *tet*(X3), *bla*_OXA-58_, *dfrA20*, *floR*, *aph(3*′*)-Ia*	SAMN14490802
				54,135	68	*mph(E)*, *msr(E)*	
GX5	*A. towneri*	2,630,890	41.53	178,808	202	*sul2*, *mph(E)*, *msr(E)*, *tet*(X3), *bla*_OXA-58,_ *dfrA20*, *floR*, *aph(3*′*)-Ia*	SAMN14490803
GX7	*A. towneri*	2,841,560	41.22	178,739	199	*sul2*, *erm(B)*, *mph(E)*, *msr(E)*, *tet*(X3), *bla*_OXA-58_, *floR*, *aph(3*′*)-Ia*	SAMN14490804

**Figure 1:**
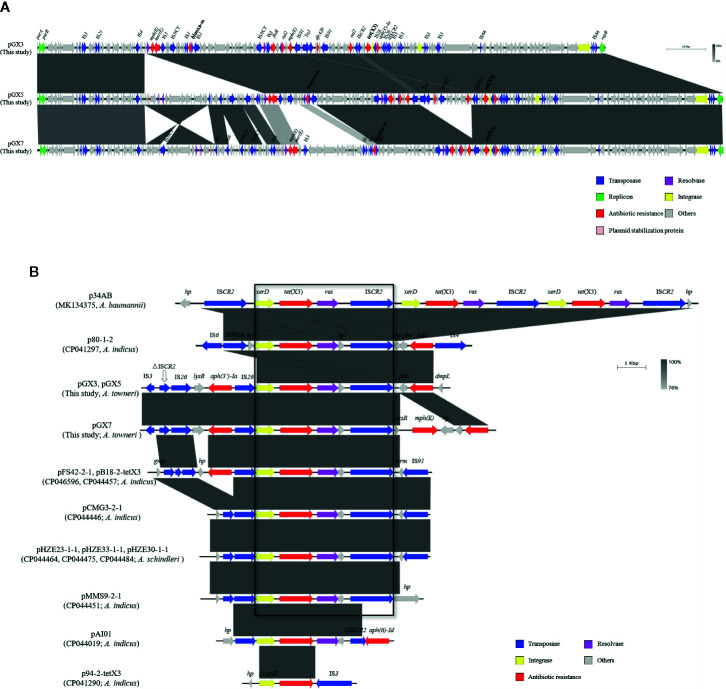
**(A)** Sequence comparison of the *tet*(X3)-harboring plasmids. Comparison of plasmids pGX3, pGX5, and pGX7. Corresponding alignments are shown in gray. Arrows and triangles represent genes of different functional categories (dark blue: transposase; green: replicon protein; red: antibiotic resistance; pink: resolvase; golden: integrase; gray: other functions). **(B)** Comparison of plasmids in this study with 11 published plasmids. Large box contains the common sequences in 10 plasmids, including the *xerD*–*tet*(X3)–*resolvase*–ΔIS*CR2* genes. Corresponding alignments are shown in gray. Arrows and triangles represent genes of different functional categories.

We analyzed the three *tet*(X3)-harboring plasmids from strains GX3, GX5, and GX7. All of them included several classes of resistance genes. As well as *tet*(X3) encoding tigecycline resistance, they contained *bla*_OXA-58_ encoding β-lactam resistance, *sul2* encoding sulphonamide resistance, *floR* encoding chloramphenicol resistance, *aph(3’)-Ia* encoding aminoglycoside resistance, and *mph(E)* and *msr(E)* encoding macrolide resistance. However, *dfrA20* encoding trimethoprim resistance was only identified in pGX3 and pGX5 (**Table 1**). Notably, all of the resistance genes were located on plasmids in these strains (**Figure 1A**).

A sequence analysis showed that the plasmids encoded the same replication protein. However, they did not belong to any known replicon type. A structural analysis of the three *tet*(X3)-harboring plasmids showed that they shared a high degree of nucleotide similarity (≥91%), suggesting that they may be derived from the same ancestral MDR plasmid backbone (**Figure 1A**).

### Analysis of the Genetic Contexts of *tet*(X3)

A comparative analysis of the genetic contexts of *tet*(X3) was performed of the plasmids detected in this study and a series of *tet*(X3)*-*harboring plasmids from *Acinetobacter* spp. recorded in the National Center for Biotechnology Information (NCBI) database. Eleven identified and annotated plasmids from the database were included (**Figure 1B**): p34AB (MK134375) from *A. baumannii*, pHZE23-1-1 (CP044464), pHZE30-1-1 (CP044484), pHZE33-1-1 (CP044475) from *A. schindleri*, p80-1-2 (CP041297), pFS42-2-1 (CP046596), pB18-2-tetX3 (CP044457), pCMG3-2-1 (CP044446), pMMS9-2-1 (CP044451), pAI01 (CP044019), and p94-2-tetX3 (CP041290) from *A. indicus*. To understand the evolutionary relationships among these *tet*(X3)*-*harboring plasmids, a phylogenetic tree was constructed based on the sequences of the *repB* genes in the plasmids (**Figure S1**). During the comparative analysis, pGX3, pGX5, and pGX7 exhibited a high degree of nucleotide similarity (91%–92% nucleotide identity and 90.11%–90.37% coverage) with eight plasmids, including pFS42-2-1, pB18-2-tetX3, pCMG3-2-1, pMMS9-2-1 and pAI01 from *A. indicus*, pHZE23-1-1, pHZE30-1-1 and pHZE33-1-1 from *A. schindleri*. The length of the eight plasmids ranged from 110-kb to 179-kb. Interestingly, no *repB*-nucleotide similarity exhibited between the plasmids in our study and the p34AB (278-kb in length) from *A. baumannii*, p80-1-2 (54-kb) and p94-2-tetX3 (42-kb) from *A. indicus*.

An analysis of their genetic environments showed that the *tet*(X3) genes were frequently located between the integrase (*xerD*) and resolvase genes. This implies that *xerD*-*tet*(X3)*-resolvase* may play an important role in the process of gene transfer. Furthermore, the *tet*(X3) gene may be transferred by IS*CR2*, which located downstream of *xerD*-*tet*(X3)*-resolvase* in all the plasmids, except p94-2-tetX3. It is interesting to note that the p34AB and p80-1-2 contain complete copies of IS*CR2* on each side of *xerD*-*tet*(X3)*-resolvase* (**Figure 1B**). Moreover, a truncated transposase (ΔIS*CR2*) (**Figure S2**) was located upstream from *xerD*-*tet*(X3) or IS*26-xerD-tet*(X3) in all plasmids (except p94-2-tetX3), which implies that the complete IS*CR2* may have existed previously. IS*CR2* belongs to the IS*91* family, which is transposed by an RC transposition mechanism that differs from those of other IS elements (Toleman and Walsh, 2010). It has been demonstrated that a 4609-bp circular intermediate occurs in p34AB (He et al., 2020b). IS*91* was previously considered a rare element in the transfer of resistance genes, but it has been increasingly detected in recent studies (Bello-López et al., 2019).

**Figure 2:**
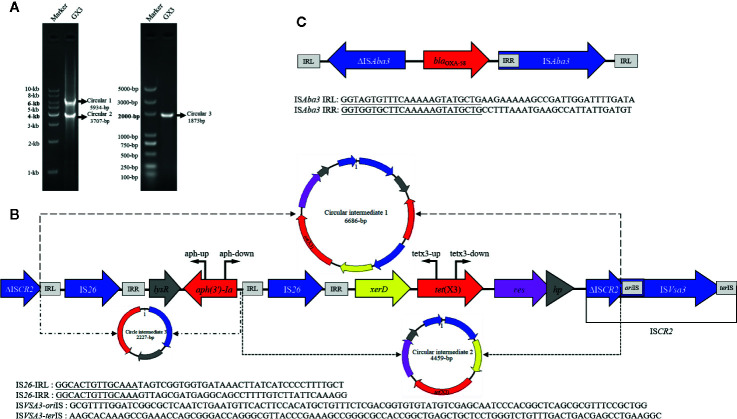
**(A)** Left: gel image of PCR products (circular 1 and circular 2) generated with the tetx3-up and tetx3-down primers. DNA ladder (1 kb) marker is used as the size standard. Right: PCR product (circular 3) generated with the aph-up and aph-down primers. DL5000 DNA Marker is used as the size standard. **(B)** Linear diagram of the genetic context of *tet*(X3) in the plasmids. The region of the circular intermediates is indicated with a dotted line and they are shown as circular graphs (circular intermediate 1, 6686 bp; circular intermediate 2, 4459 bp; circular intermediate 3, 2227 bp). Locations of primers used to map the targeted circular intermediates are shown with bent arrows. **(C)** Linear diagram of the genetic contexts of *bla*_OXA-58_ in the plasmids isolated in this study.

Previous studies have been shown that IS*CR2* can move additional sequences located upstream from the transposase gene (Toleman et al., 2006). We used inverse PCR to examine whether this region could be mobilized by RC replication (**Figure 2A**). The inverse PCR primers used have been reported previously (He et al., 2020b). Interestingly, 5934-bp and 3707-bp amplicons were generated and a sequence analysis showed that both of them lacked the complete IS*CR2* (IS*26-LysR*-*aph(3’)-Ia*-IS*26*-*xerD*-*tet*(X3)-*resolvase*-*hp*-ΔIS*CR2* (6686-bp) and IS*26*-*xerD*-*tet*(X3)-*resolvase*-*hp*-ΔIS*CR2* (4459-bp)) (**Figure 2B**). It is notable that *ori*IS and *ter*IS were not detected in ΔIS*CR2.* We also detected a minicircle containing *aph(3’)-Ia* (IS*26-LysR*-*aph(3’)-Ia* (2227-bp)) with an inverse PCR assay (aph-up: 5′-CGGTTTGGTTGATGCGAGTG-3′, aph-down:5′-GATTGTCGCACCTGATTGCC-3′) (**Figure 2B**). These results suggest that a circular intermediate was generated by IS*26*, which can disseminate antibiotic resistance genes *via* a translocatable unit (TU) containing only one copy of IS*26* (Harmer and Hall, 2015). It is tempting to speculate that the 3′-end (GAAC) of ΔIS*CR2* is a specific target sequence in the TU in which 5′-GAAC or 5′-GTTC is the insertion target sequence in IS*91* transfer (Toleman et al., 2006). IS*26* also frequently occurs upstream from of *tet*(X3) in published plasmids recorded in the NCBI, and may increase the risk of *tet*(X3) dissemination (**Figure 1B**). The mechanism involved warrants further research.

### Analysis of the Genetic Context of *bla*_OXA-58_

We also identified the carbapenemase gene, *bla*_OXA-58_, in these isolates. The *bla*_OXA-58_ gene was all present in the *tet*(X3)-harboring plasmids, pGX3, pGX5, and pGX7 in our study. Further examination of the *bla*_OXA-58_ region revealed that a truncated IS*Aba3* element was located upstream of *bla*_OXA-58_ and a complete IS*Aba3* element on downstream in all plasmids (**Figure 2C**). This is consistent with a previous report that the *bla*_OXA-58_ gene was transferred by IS*Aba3* (Matos et al., 2019). It is notable that the isolates were resistant to imipenem (MIC, 8–16 mg/L), but they were susceptible to meropenem (MIC, 0.12–2 mg/L) (**Table S1**). It may be due to *bla*_OXA-58_ hydrolyzes carbapenems weakly and often expresses poorly. Moreover, the insertion of an upstream IS*Aba3* can enhance expression of the *bla*_OXA-58_, but it is truncated in these strains (Hamidian and Nigro, 2019). Overall, our findings support the notion that the co-occurrence of *bla*_OXA-58_ and *tet*(X3) strengthens the risk of the dissemination of these plasmids.

## Conclusion

To the best of our knowledge, this is the first report of the co-occurrence of *tet*(X3) and *bla*_OXA-58_ in plasmids identified from *A. towneri*. What is remarkable here is that, although *A. towneri* is an environmental bacterium, it was detected in the faeces of swine. pGX3 has a novel plasmid backbone containing MDR genes, which are similar to the backbone structures of pGX5 and pGX7, suggesting that they may be derived from the same ancestral MDR plasmid backbone. Like IS*CR2*, IS*26* has been proved that it can generate a circular intermediate to transfer *tet*(X3), providing antibiotic resistance genes with a highly mobile genetic vehicle, although the mechanisms involved require further research.

## Data Availability Statement

The datasets presented in this study can be found in online repositories. The names of the repository/repositories and accession number(s) can be found at: (https://www.ncbi.nlm.nih.gov/), SRR11454657 (https://www.ncbi.nlm.nih.gov/), SRR11454656 (https://www.ncbi.nlm.nih.gov/), SRR11454655.

## Author Contributions

JM, JW, and ZY conceived and designed the study. JF, YL, BY, and XW acquired the data. JM, RL, LB, and TH drafted the manuscript. JW critically revised the manuscript. All authors contributed to the article and approved the submitted version.

## Funding

This work was jointly supported by the National Natural Science Foundation of China (grant numbers 31702294, 31930110 and 31802230); China Postdoctoral Science Foundation (grant numbers 2018T111114); Natural Science Basic Research Plan in Shaanxi Province of China (Grant No. 2019JQ-281) and China Agriculture Research System (grant numbers CARS-39-14). The funders had no role in study design, data collection and analysis, decision to publish, or preparation of the manuscript.

## Conflict of Interest

The authors declare that the research was conducted in the absence of any commercial or financial relationships that could be construed as a potential conflict of interest.

## Supplementary Material

Supplementary Figure 1 Neighbour-joining tree based on the plasmid replication initiator protein gene sequences, showing the phylogenetic positions of the *tet*(X3)-harboring plasmids from strains GX3, GX5, and GX7 (indicated by ●) and from some other representative related taxa. Bootstrap values (expressed as percentages of 1,000 replications) greater than 50% are shown at the branch points. Scale bar, 0.05 substitutions per nucleotide position.

Supplementary Figure 2 Nucleotide sequence of ΔIS*CR2*. Position of the transposase gene is indicated by green dots under the sequence. Predicted amino acid sequence of the transposase is given with the single-letter amino acid code, placed below the right nucleotide of each codon. Specific target sequence (-GAAC) is indicated with a black box.
