# Supplementary figures and images for: Characterization of Three Porcine Acinetobacter towneri Strains Co-Harboring tet(X3) and bla OXA-58
Source: Front Cell Infect Microbiol. 2020 Dec 10;10:586507. doi: 10.3389/fcimb.2020.586507 (PMC7758954; doi:10.3389/fcimb.2020.586507)

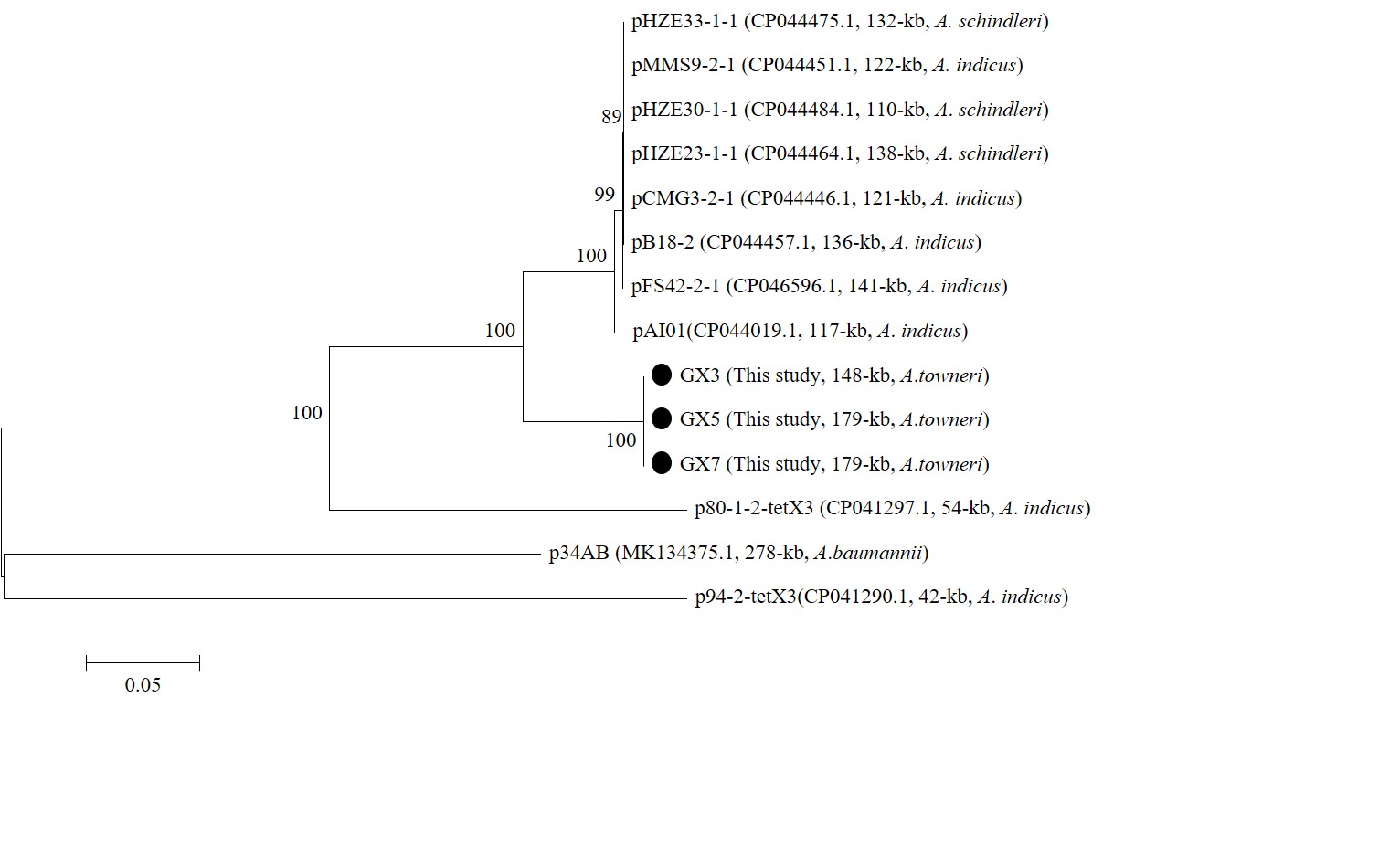

Supplement: Supplementary Figure 1 — Neighbour-joining tree based on the plasmid replication initiator protein gene sequences, showing the phylogenetic positions of the tet(X3)-harboring plasmids from strains GX3, GX5, and GX7 (indicated by ●) and from some other representative related taxa. Bootstrap values (expressed as percentages of 1,000 replications) greater than 50% are shown at the branch points. Scale bar, 0.05 substitutions per nucleotide position. [file Image_1.jpeg]

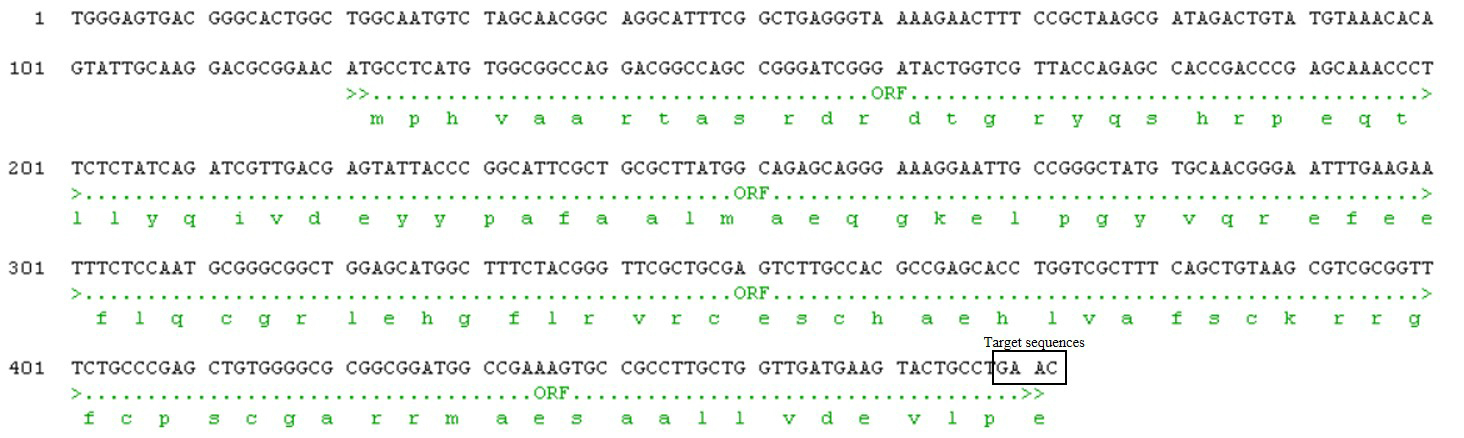

Supplement: Supplementary Figure 2 — Nucleotide sequence of ΔISCR2. Position of the transposase gene is indicated by green dots under the sequence. Predicted amino acid sequence of the transposase is given with the single-letter amino acid code, placed below the right nucleotide of each codon. Specific target sequence (-GAAC) is indicated with a black box. [file Image_2.jpeg]
