# Supplementary material for: Characterization of Three Porcine Acinetobacter towneri Strains Co-Harboring tet(X3) and bla OXA-58
Source: Front Cell Infect Microbiol. 2020 Dec 10;10:586507. doi: 10.3389/fcimb.2020.586507 (PMC7758954; doi:10.3389/fcimb.2020.586507)
Supplement: Supplementary file 3 [file Table_1.docx]

Supplementary Material

Table S1. Antimicrobial susceptibility test of *A. towneri* strains in this study.

| Strain |  | MIC (mg/L) | | | | | | | | | | | | | | |
| --- | --- | --- | --- | --- | --- | --- | --- | --- | --- | --- | --- | --- | --- | --- | --- | --- |
|  | AMP | AUG | GEN | SPE | TET | FFC | SUL | SULF | CEF | CAZ | ENR | OFL | IMP | MER | TIG | COL |
| GX3 | 32 | 32/16 | 0.5 | 512 | 16 | 64 | 512 | 16 | 16 | 64 | 0.5 | 4 | 8 | 0.12 | 16 | 0.12 |
| GX5 | 256 | 64/32 | 0.25 | 512 | 16 | 64 | 512 | 8 | 32 | 64 | 1 | 8 | 16 | 2 | 16 | 0.12 |
| GX7 | 64 | 32/16 | 0.5 | >512 | 16 | 32 | 512 | 8 | 8 | 64 | 2 | 8 | 16 | 0.5 | 16 | 0.12 |

AMP: ampicillin; AUG, amoxicillin-clavulanate; GEN, gentamicin; SPE, spectinomycin; TET, tetracycline; FFC, florfenicol; SUL, sulfisoxazole; SULF, sulfamethoxazole; CEF, ceftiofur; CAZ, ceftazidime; ENR, enrofloxacin; OFL, ofloxacin; IMP, imipenem; MER, meropenem; TIG, tigecycline; COL, colistin.
